# Combined effect of serum carcinoembryonic antigen and hepatic steatosis on new-onset ischemic heart disease among middle-aged and older Korean adults: a cohort study

**DOI:** 10.3389/fnut.2023.1275450

**Published:** 2023-10-10

**Authors:** Ji Won Kwak, Yong Jae Lee, Byoungjin Park, Dong Hyuk Jung

**Affiliations:** Yonsei University Health System, Seoul, Republic of Korea

**Keywords:** carcinoembryonic antigen, hepatic steatosis, ischemic heart disease, middle-aged and older adults, cohort study

## Abstract

**Background:**

Carcinoembryonic antigen (CEA) is a commonly used tumor marker in cancer screening. However, it has also been associated with metabolic alterations. Hepatic steatosis, the accumulation of fat in liver cells, is associated with various cardiovascular risk factors. This study investigated the risk of ischemic heart disease (IHD) in individuals with elevated CEA levels, hepatic steatosis, and their co-occurrence.

**Methods:**

The study cohort comprised 5,580 Korean adults who underwent health examinations between November 2006 and June 2010. Data regarding baseline CEA levels, hepatic steatosis status, and development of IHD were collected. Hepatic steatosis was defined as more than two findings: deep attenuation, vascular blurring, and increased liver echogenicity on abdominal ultrasound. Participants were divided into four groups based on their CEA and hepatic steatosis status: no hepatic steatosis and low CEA (group 1), no hepatic steatosis and elevated CEA (group 2), low CEA and hepatic steatosis (group 3), and elevated CEA and hepatic steatosis (group 4).

**Results:**

A total of 226 (4.1%) participants developed IHD during the follow-up period. Participants with elevated CEA levels and hepatic steatosis (group 4) had the highest cumulative incidence of IHD in comparison to other groups (*p* < 0.001). The combined effect of elevated CEA levels and hepatic steatosis showed significantly greater area under the receiver operating characteristic curve than hepatic steatosis alone (*p* < 0.001). Furthermore, participants with elevated CEA and hepatic steatosis (group 4) had higher risk of developing IHD compared to those with low CEA and no hepatic steatosis (group 1) (hazard ratio: 1.63, 95% confidence interval: 1.04–2.55, *p* = 0.034).

**Conclusion:**

Co-occurrence of elevated CEA levels and hepatic steatosis increases the risk of IHD. Comprehensive risk assessment is crucial to guide interventions and improve cardiovascular health in individuals with both the conditions.

## 1. Introduction

Carcinoembryonic antigen (CEA) is a commonly used tumor marker in various types of cancer. It serves as a valuable tool for diagnosis, prognosis, and monitoring of treatment responses (1–4). However, recent evidence suggests that CEA levels may have broader implications beyond cancer detection. Several studies have reported associations between elevated CEA levels and diseases other than cancer, including components of metabolic syndrome such as hypertension, diabetes, hyperlipidemia, obesity, and insulin resistance (5, 6). Furthermore, CEA has been associated with atherosclerotic diseases, including carotid artery and coronary heart diseases (7, 8). Elevated CEA levels may reflect the underlying inflammatory process and endothelial dysfunction, thereby contributing to the development and progression of atherosclerosis. Consequently, it is plausible that elevated CEA levels could serve as a potential biomarker for identifying individuals at risk for ischemic heart disease (IHD).

Hepatic steatosis, which is characterized by accumulation of fat in the hepatocytes, has emerged as a significant health concern due to its association with various adverse health outcomes. Hepatic steatosis has been implicated in the pathogenesis of IHD through multiple mechanisms, including endothelial dysfunction, insulin resistance, increased inflammation, and elevated oxidative stress (9). Previous studies have demonstrated that hepatic steatosis independently contributes to adverse cardiovascular outcomes, even after accounting for traditional cardiovascular risk factors (10). Therefore, the risk of developing IHD may increase further if elevated CEA levels and hepatic steatosis are present simultaneously. However, this has not been well-established.

We hypothesized that the concurrent effect of elevated CEA levels and hepatic steatosis could lead to a better prediction of the risk of developing IHD. Therefore, this study aimed to investigate the risk of IHD development in patients with elevated CEA levels, hepatic steatosis, and their co-occurrence.

## 2. Materials and methods

### 2.1. Study design and participants

**Figure 1:**
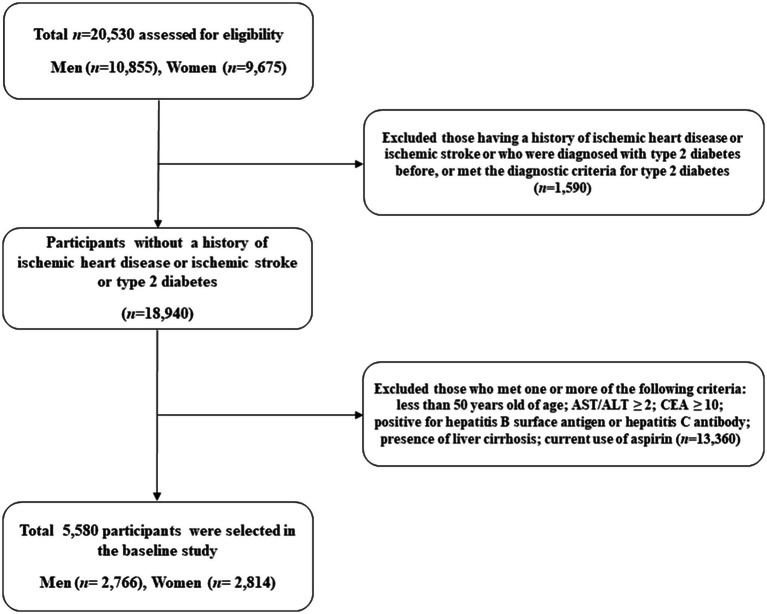
Flowchart depicting selection of the study population.

We used the Health Risk Assessment Study and Health Insurance Review and Assessment Service data (HERAS-HIRA dataset) to explore the surrogate indicators for IHD among Korean adults (11, 12). The study cohort comprised 20,530 sequentially enrolled participants who voluntarily visited the Health Promotion Centre, Gangnam Severance Hospital, Yonsei University College of Medicine, for health examination between November 2006 and June 2010. Among the initially assessed participants, we excluded 1,590 (7.7%) individuals with a history of IHD or ischemic stroke, previous diagnosis of type 2 diabetes, or fasting plasma glucose (FPG) level ≥ 126 mg/dL. Participants who met even one of the following criteria were also excluded from the study: younger than 50 years of age, CEA ≥ 10.0 ng/mL, aspartate aminotransferase (AST)/ alanine aminotransferase (ALT) ≥ 2.0, positive for hepatitis B surface antigen or hepatitis C antibody, presence of liver cirrhosis, and current use of aspirin (*n* = 13,360). After these exclusions, a total of 5,580 participants (2,766 men and 2,814 women) were included in the final analysis (Figure 1).

### 2.2. Data collection

All the participants completed a questionnaire on their lifestyle and medical history. Self-reported cigarette smoking, alcohol consumption, and physical activity data were obtained from the questionnaires. Smoking status was categorized as non-smoker, ex-smoker, or current smoker. Regular alcohol consumption was defined as ≥140 grams of alcohol consumption per week. Participants were enquired about their physical exercise levels, and regular exercise was defined as moderate intensity physical activity ≥ three times per week (13–15). Systolic and diastolic blood pressures were measured on the patient’s right arm using a standard mercury sphygmomanometer with the participant in a sitting position after 10 min of rest (Baumanometer, W.A. Baum Co., Inc., Copiague, NY, USA). All blood samples were obtained from the antecubital vein after overnight fasting for 12 h. Fasting plasma glucose, total cholesterol, triglyceride, high-density lipoprotein cholesterol, and liver enzymes were measured by enzymatic methods using a Hitachi 7,600 automated chemistry analyzer (Hitachi Co., Tokyo, Japan). Serum CEA levels were measured via the chemiluminescence immunoassay (CLIA) method using a Unicel DXI800 analyzer (Beckman-Coulter, Chaska, MN, United States). C-reactive protein concentrations were measured using the Roche/Hitachi 912 System (Roche Diagnostics, Indianapolis, IN, USA) and latex-enhanced immunoturbidimetric method. Hypertension was defined as systolic blood pressure ≥ 140 mmHg, diastolic blood pressure ≥ 90 mmHg, or current use of anti-hypertensive drugs (16).

### 2.3. Abdominal ultrasonography

Liver ultrasonography was performed by experienced radiologists who were blinded to the laboratory and clinical data using a 3.5-MHz transducer (HDI 5000, Philips, Bothell, WA, United States). No information was available regarding this study. Patients were diagnosed with fatty liver if more than two of the following three findings were detected: deep attenuation, vascular blurring, and increased liver echogenicity (17).

### 2.4. Outcomes

The study outcome assessed was IHD, which consisted of angina pectoris (ICD-10 code I20) or acute myocardial infarction (ICD-10 code I21) that developed after initial study enrollment. To define baseline and post-survey outcomes, we linked a personal 13-digit identification number that was assigned to each participant with HIRA data in South Korea between November 2006 and December 2010.

### 2.5. Statistical analysis

We classified the participants into low and elevated CEA on the basis of 50 percentiles (CEA = 1.6 ng/mL) after excluding high CEA levels (18). The study participants were then divided into four groups: no hepatic steatosis and low CEA (group 1); no hepatic steatosis and elevated CEA levels (group 2); low CEA and hepatic steatosis (group 3); and elevated CEA levels and hepatic steatosis (group 4). The baseline characteristics of the participants in each group were compared using analysis of variance (ANOVA) for continuous variables and Chi-square test for categorical variables. We have used box plots and the Kolmogorov–Smirnov test to evaluate the distribution of the variables. Log transformation and analysis were performed for C-reactive protein that does not exhibit a normal distribution. Age and sex-adjusted survival curves were used to estimate the cumulative incidence of IHD in each group. After setting the first group as the reference group, the hazard ratios (HRs) and 95% confidence intervals (CIs) for IHD were calculated using multivariate Cox proportional hazards regression models after adjusting for potential confounding variables. Pairwise comparisons of receiver operating characteristic (ROC) curves were used to compare the area under the ROC curve (AUC) for IHD incidence based on CEA status and presence of hepatic steatosis. To assess the features of the combined effect, we compared the HR according to the presence of each individual and among all participants. All the analyses were performed using SAS version 9.4 (SAS Institute Inc., Cary, NC, United States). All statistical tests were two-sided, and statistical significance was set at *p* < 0.05.

## 3. Results

### 3.1. Baseline characteristics

**Figure 2:**
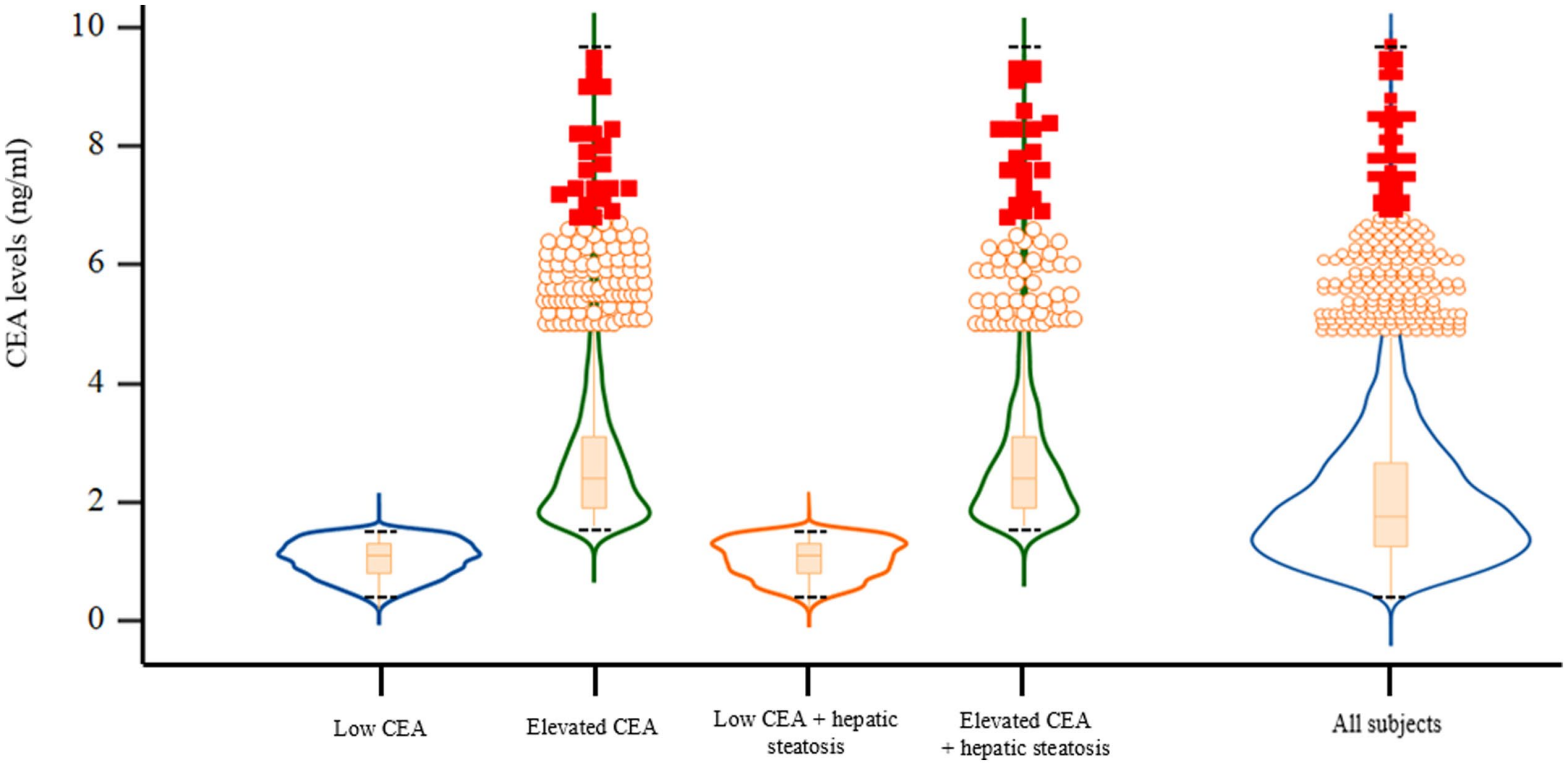
Violin plots with embedded box plots according to CEA and hepatic steatosis. CEA, carcinoembryonic antigen. Black dotted lines indicate the maximum and minimum CEA values of each group.

We have demonstrated the baseline characteristics of the study population (*n* = 5,580; 2,766 men and 2,814 women) according to serum CEA levels and hepatic steatosis status (Table 1; Figure 2). The mean age and BMI of the study participants were 57.0 ± 6.3 years and 23.7 ± 2.7 kg/m^2^, respectively. The mean CEA levels were 1.95 ± 1.23 ng/mL for all participants and 1.04 ± 0.31 (min 0.2, max 1.5), 2.72 ± 1.17 (min 1.6, max 9.5), 1.04 ± 0.32 (min 0.2, max 1.5), 2.73 ± 1.22 (min 1.6, max 9.3) ng/ml for the first, second, third, and fourth groups, respectively. The mean values of mean blood pressure, liver enzymes, fasting plasma glucose, triglyceride, and C-reactive protein levels were highest in the group with elevated CEA levels and hepatic steatosis (group 4). The proportion of alcohol consumption habit and hypertension were also the highest in group 4.

**Table 1:** Baseline characteristics of the study population.

Variables	Group 1	Group 2	Group 3	Group 4	*p* value	
	Low CEA (*n* = 1,572)	Elevated CEA (*n* = 1,862)	Low CEA + hepatic steatosis (*n* = 986)	Elevated CEA + hepatic steatosis (*n* = 1,160)		Post hoc^a^
Age (years)	55.9 ± 5.7	57.6 ± 6.8	56.5 ± 5.7	57.7 ± 6.5	< 0.001	a,c,d,f
Male sex (%)	41.6	41.1	69.1	60.5	< 0.001	-
Body mass index (kg/m^2^)	22.8 ± 2.4	22.9 ± 2.5	25.0 ± 2.5	25.2 ± 2.4	< 0.001	b,c,d,e
Systolic blood pressure (mmHg)	122.6 ± 15.8	123.9 ± 15.7	127.5 ± 14.5	129.5 ± 15.4	< 0.001	b,c,d,e,f
Diastolic blood pressure (mmHg)	76.6 ± 9.8	77.4 ± 9.8	79.9 ± 9.2	81.4 ± 9.6	< 0.001	b,c,d,e,f
Mean blood pressure (mmHg)	91.9 ± 11.4	92.9 ± 11.3	95.7 ± 10.5	97.4 ± 11.1	< 0.001	b,c,d,e,f
CEA (ng/ml)	1.04 ± 0.31	2.72 ± 1.17	1.04 ± 0.32	2.73 ± 1.22	< 0.001	a,c,d,f
Fasting plasma glucose (mg/dl)	92.0 ± 9.1	92.1 ± 10.1	96.8 ± 10.2	97.3 ± 10.7	< 0.001	b,c,d,e
Total cholesterol (mg/dl)	196.5 ± 34.2	195.5 ± 32.8	203.8 ± 34.2	204.0 ± 34.4	< 0.001	b,c,d,e
Triglyceride (mg/dl)	106.2 ± 52.1	111.3 ± 59.4	157.0 ± 83.5	164.4 ± 96.2	< 0.001	b,c,d,e
HDL cholesterol (mg/dl)	55.5 ± 12.5	55.8 ± 13.6	47.9 ± 10.1	48.1 ± 11.1	< 0.001	b,c,d,e
Aspartate aminotransferase (IU/L)	21.2 ± 11.9	22.1 ± 8.6	23.5 ± 8.8	24.6 ± 9.9	< 0.001	b,c,d,e,f
Alanine aminotransferase (IU/L)	19.3 ± 17.2	20.0 ± 11.5	26.7 ± 15.0	28.0 ± 16.6	< 0.001	b,c,d,e
Log C-reactive protein (mg/L)	−0.55 ± 1.07	−0.39 ± 1.09	−0.02 ± 0.99	0.06 ± 1.02	< 0.001	a,b,c,d,e
Current smoker (%)	6.8	22.9	9.6	25.3	< 0.001	–
Alcohol drinking (%)	27.4	35.4	31.1	39.7	< 0.001	–
Regular exercise (%)	43.4	43.1	35.8	36.5	< 0.001	–
Hypertension (%)	26.2	28.5	38.4	43.0	< 0.001	–

CEA, carcinoembryonic antigen; HDL, High density-lipoprotein. *p* values were calculated using one-way ANOVA test or Pearson’s chi-square test.

a Post hoc analysis with Bonferroni method: a, Q1 versus Q2; b, Q1 versus Q3; c, Q1 versus Q4; d, Q2 versus Q3; e, Q2 versus Q4; and f, Q3 versus Q4.

### 3.2. IHD incidence and AUC comparison

**Figure 3:**
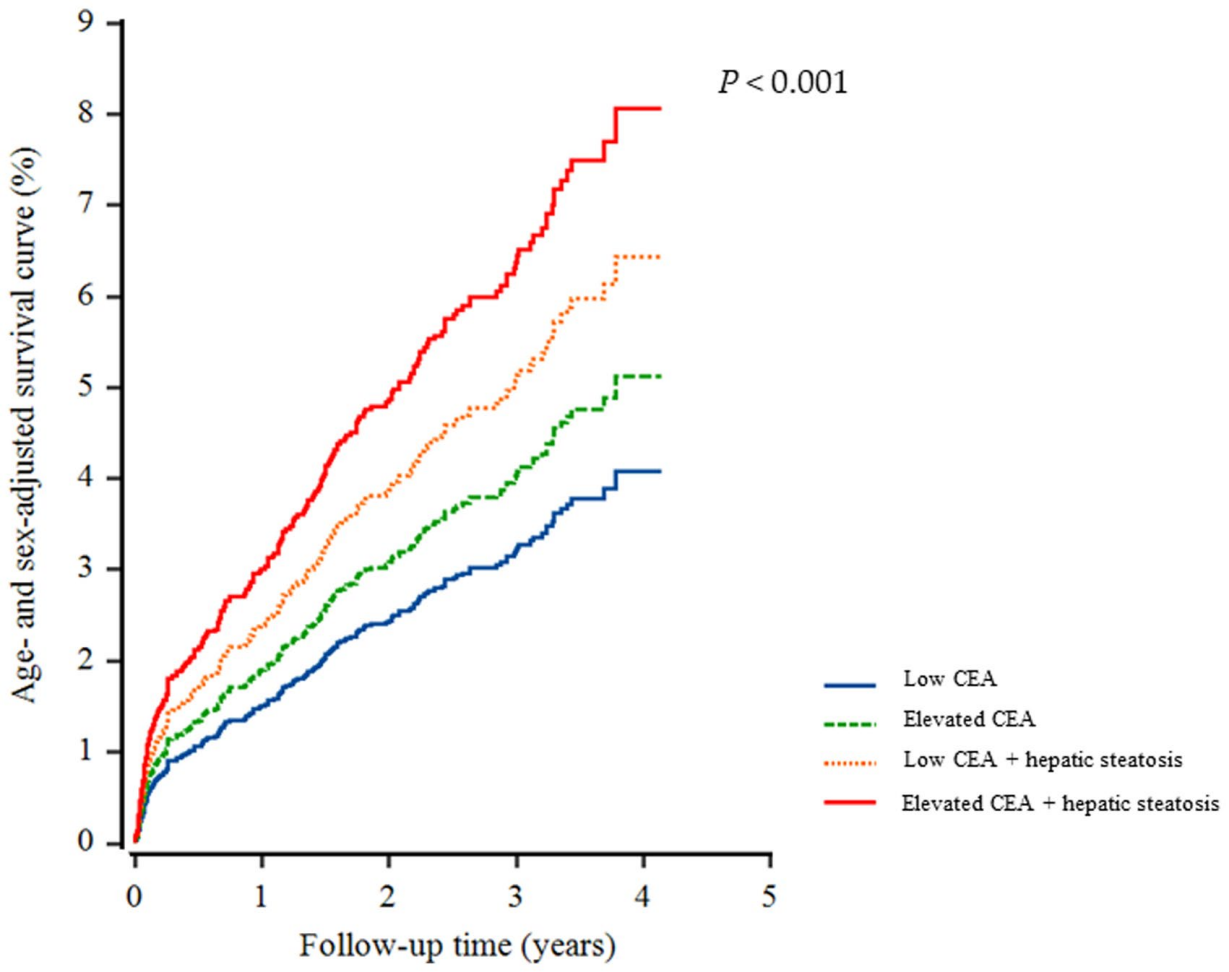
Cox regression survival curve for incidence of ischemic heart disease.

**Figure 4:**
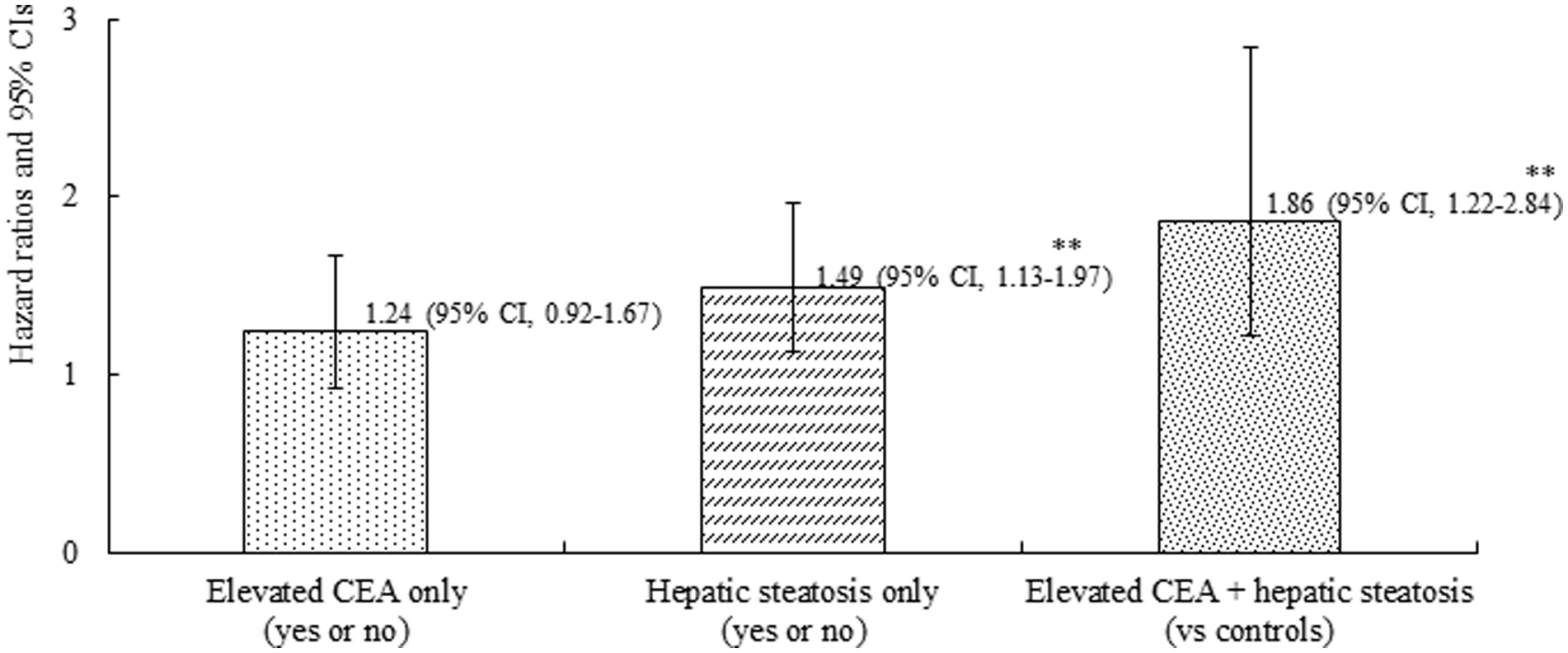
Combined effect of carcinoembryonic antigen and hepatic steatosis on incident ischemic heart disease. Age-and sex-adjusted Cox proportional hazards regression model analysis. ^**^p < 0.001.

A total of 226 individuals (4.1%) developed IHD during the follow-up period. Furthermore, group 4 exhibited the highest cumulative incidence of IHD up to 50 months after adjusting for age and sex (*p* < 0.001) (Figure 3). The incidence rates per 1,000 person-years were 11.2, 16.1, 19.2, and 26.3 in the four groups, respectively. Figure 4 shows that elevated CEA levels and hepatic steatosis are additive features for the development of IHD. Using pairwise comparison of ROC analyses of incident IHD, the AUC of the groups classified according to elevated CEA levels and hepatic steatosis was significantly higher than that of the group classified based on the presence of hepatic steatosis (*p* < 0.001). The AUC, sensitivity, and specificity of the groups according to elevated CEA with hepatic steatosis were 0.589, 50.0, and 62.0%, respectively (Table 2).

**Table 2:** Elevated carcinoembryonic antigen with hepatic steatosis versus elevated carcinoembryonic antigen and hepatic steatosis for predicting ischemic heart disease.

	Pairwise comparison of AUC			Ability to classify IHD			
	Difference	95% CI	*p* value	Sensitivity (%)	Specificity (%)	AUC	*p* value
Elevated CEA with hepatic steatosis vs. elevated CEA	0.032	−0.004 to 0.069	0.085				
Elevated CEA with hepatic steatosis vs. hepatic steatosis	0.029	0.012 to 0.045	< 0.001				
Elevated CEA vs. hepatic steatosis	0.003	−0.043 to 0.050	0.885				
Elevated CEA with hepatic steatosis				50.0	62.0	0.589	< 0.001
Elevated CEA				65.0	46.3	0.557	< 0.001
Hepatic steatosis				50.0	62.0	0.560	< 0.001

AUC, area under the receiver operating characteristic curve; CEA, carcinoembryonic antigen; IHD, ischemic heart disease.

### 3.3. Hazard ratios for incident IHD

**Figure 5:**
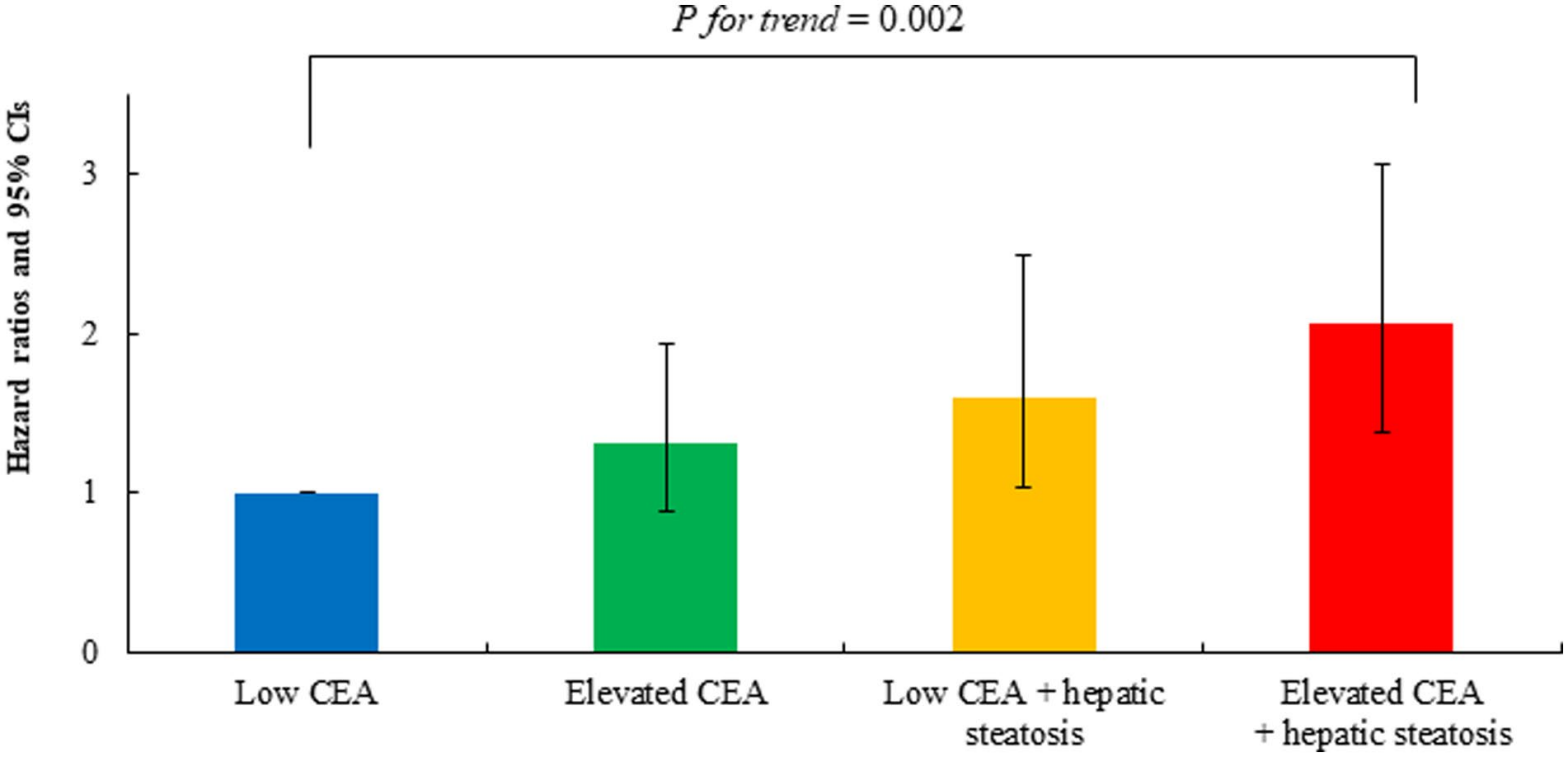
Age-and sex-adjusted hazard ratios (95% confidence intervals) for incident ischemic heart disease according to carcinoembryonic antigen and hepatic steatosis.

Table 3 shows the results of multivariate Cox proportional hazards regression analysis for prediction of IHD according to CEA levels and hepatic steatosis. Compared to the referent group with low CEA, the HRs for IHD were 1.31 (95% CI, 0.88–1.94) in group 2, 1.60 (95% CI, 1.03–2.49) in group 3, and 2.06 (95% CI, 1.38–3.07) in group 4, after adjusting for age and sex (Model 1, Table 3; Figure 5). Similarly, positive associations were found after additionally adjusting for body mass index, smoking status, alcohol consumption, regular exercise, mean arterial blood pressure, fasting plasma glucose, high-density lipoprotein cholesterol, and anti-hypertensive medication (Model 3). The corresponding adjusted HR for group 4 versus group 1 was 1.63 (95% CI, 1.04–2.55) (*p* = 0.034).

**Table 3:** Hazard ratios and 95% confidence intervals for incident ischemic heart disease.

		Group 1	Group 2	Group 3	Group 4	*P* for trend
		Low CEA	Elevated CEA	Low CEA + hepatic steatosis	Elevated CEA + hepatic steatosis	
New cases of ischemic heart disease, *n*		39	74	40	73	
Mean follow-up, years		2.2 ± 1.1	2.5 ± 1.1	2.1 ± 1.0	2.4 ± 1.1	
Pearson-years of follow-up		3,477	4,587	2085	2,775	
Incidence rate/1000 person-years		11.2	16.1	19.2	26.3	
Model 1	HR (95% CI)	1.00 (reference)	1.31 (0.88–1.94)	1.60 (1.03–2.49)	2.06 (1.38–3.07)	0.002
	*P* value	–	0.179	0.038	< 0.001	
Model 2	HR (95% CI)	1.00 (reference)	1.26 (0.83–1.91)	1.39 (0.85–2.26)	1.78 (1.14–2.79)	0.074
	*P* value	–	0.277	0.191	0.011	
Model 3	HR (95% CI)	1.00 (reference)	1.28 (0.84–1.94)	1.24 (0.76–2.03)	1.63 (1.04–2.55)	0.191
	*P* value	–	0.245	0.392	0.034	

Model 1: adjusted for age and sex. Model 2: adjusted for age, sex, body mass index, smoking status, alcohol consumption, and regular exercise. Model 3: adjusted for age, sex, body mass index, smoking status, alcohol consumption, regular exercise, mean arterial blood pressure, fasting plasma glucose, high-density lipoprotein cholesterol, and anti-hypertensive medication.

## 4. Discussion

This study aimed to investigate the association between elevated CEA levels and hepatic steatosis, and their combined effects on the risk of IHD. To achieve this, we categorized 5,580 participants into four groups on the basis of their status of elevated CEA, hepatic steatosis, or both, and analyzed the association of each group with the risk of IHD.

Some studies have demonstrated a significant association among CEA levels, hepatic steatosis, and development of cardiovascular disease (7, 8, 10, 19). Patients with acute coronary syndrome showed significantly elevated CEA levels compared to the control groups (8). Also, elevated CEA levels have been associated with carotid atherosclerosis, suggesting its involvement in the development of atherosclerotic diseases (7). However, there was no positive association between CEA and cardiovascular disease, in the 2017–2018 National Health and Nutrition Examination Survey (20). The novelty of this study was to investigate the risk of IHD in patients with elevated CEA levels, hepatic steatosis, and their co-occurrence. During a follow-up period of approximately 4 years, individuals with both elevated CEA levels and hepatic steatosis showed a significantly higher risk of developing IHD in comparison to those belonging to other groups. Furthermore, this association persisted even after adjusting for confounding variables. Moreover, participants with elevated CEA levels and hepatic steatosis displayed a significantly greater AUC than those with hepatic steatosis alone.

The mechanisms underlying the association between serum CEA levels and atherosclerotic disease have been suggested in previous studies (7, 21). Serum CEA levels are linked to metabolic syndrome and insulin resistance, which increase the risk of atherosclerotic vascular disease (22). Moreover, CEA can stimulate the release of proinflammatory cytokines, leading to the expression of adhesion molecules on endothelial cells and potentially facilitating early atherosclerosis (7, 23). Additionally, elevated serum CEA levels have also been observed in chronic inflammatory disorders, which may further increase the risk of cardiovascular diseases (24, 25). Similarly, an association between hepatic steatosis and cardiovascular disease has been suggested. Insulin resistance plays a significant role in the association between hepatic steatosis and cardiovascular disease. Studies have consistently demonstrated a correlation between hepatic steatosis and insulin resistance, with higher levels of hepatic fat being associated with increased insulin resistance (26–28). It has also been proposed that hepatic steatosis may occur as a consequence of primary generalized insulin resistance (29–31). Furthermore, hepatic steatosis is associated with proatherogenic conditions such as metabolic alterations and chronic low-grade inflammation, both of which may contribute to developing IHD (32). A previous study reported a relationship between CEA and hepatic steatosis, indicating that serum CEA levels gradually increase with the severity of hepatic steatosis (33). This study suggests that chronic inflammation, decreased hepatic clearance, and direct secretion from fat stores could serve as mechanisms linking CEA levels to hepatic steatosis. Co-occurrence of elevated CEA levels and hepatic steatosis may augment insulin resistance and metabolic disturbances, leading to an increased risk of IHD. Furthermore, the interaction between elevated CEA levels and hepatic steatosis may synergistically enhance the proinflammatory and endothelial-damaging effects, thereby promoting the development of atherosclerotic plaques and IHD.

This study had a few limitations. First, we did not fully consider the participants’ drug history, including the use of liver pills, statins, and antiplatelet medications other than aspirin and hypertension medication. Second, the baseline atherosclerotic burden was not fully examined. The presence and severity of preexisting atherosclerosis can significantly influence the development and progression of IHD. It is possible that individuals with a higher baseline atherosclerotic burden may be more susceptible to the effects of hepatic steatosis and elevated CEA on the risk of IHD. Therefore, not accounting for baseline atherosclerosis could introduce a confounding factor and limit the comprehensive understanding of the association being studied. Third, hepatic steatosis and CEA levels may vary over time, and their progression or regression may affect the development of IHD. Assessing the changes in hepatic steatosis and CEA levels during the study period could provide valuable information regarding the dynamic nature of these factors and their association with IHD risk. Individuals who experience worsening or persistent hepatic steatosis, as well as those with continuously elevated CEA levels, may have a higher risk of developing IHD in comparison to those with stable or improving conditions. Last, we did not consider some liver inflammatory conditions, such as liver fibrosis or alcohol steatohepatitis, and socioeconomic status because of insufficient alcohol surveys, and those indicators were not measured at the beginning of this study. To minimize the inclusion of alcoholic steatohepatitis, we have excluded those with AST/ALT >2. Further prospective research is warranted to elucidate the cause-and-effect link between CEA, hepatic steatosis, and IHD, considering specific liver conditions and socioeconomic status.

In conclusion, this study suggests that elevated CEA levels and hepatic steatosis, particularly when present together, are associated with an increased risk of IHD. These findings suggest that patients with hepatic steatosis and elevated CEA levels should undergo comprehensive risk assessment for IHD to address modifiable risk factors and implement appropriate interventions for risk reduction and improvement in cardiovascular health.

## Data availability statement

The raw data supporting the conclusions of this article will be made available by the authors, without undue reservation.

## Ethics statement

The studies involving humans were approved by the Institutional Review Board of Yonsei University College of Medicine. The studies were conducted in accordance with the local legislation and institutional requirements. The human samples used in this study were acquired from a by- product of routine care or industry. Written informed consent for participation was not required from the participants or the participants' legal guardians/next of kin in accordance with the national legislation and institutional requirements.

## Author contributions

JK: Conceptualization, Formal analysis, Investigation, Methodology, Writing – original draft. YL: Conceptualization, Data curation, Investigation, Methodology, Project administration, Resources, Writing – review & editing. BP: Conceptualization, Data curation, Formal analysis, Investigation, Methodology, Project administration, Supervision, Validation, Writing – review & editing. DJ: Conceptualization, Formal analysis, Investigation, Methodology, Project administration, Supervision, Validation, Writing – review & editing.
